# Health-Related Quality of Life Using the KIDSCREEN-27 Questionnaire among Adolescents with High Myopia

**DOI:** 10.3390/jcm13133676

**Published:** 2024-06-24

**Authors:** Joanna Zawistowska, Katarzyna Powierza, Jolanta Sawicka-Powierza, Justyna Macdonald, Mirosława Czerniawska, Alexandra Macdonald, Zuzanna Przystupa, Alina Bakunowicz-Łazarczyk

**Affiliations:** 1 Department of Paediatric Ophthalmology and Strabismus, Medical University of Bialystok, ul. Waszyngtona 17, 15-274 Bialystok, Poland; alina.bakunowicz-lazarczyk@umb.edu.pl; 2 Department of Diabetology and Internal Medicine, Medical University of Warsaw, ul. S. Banacha 1A, 02-097 Warsaw, Poland; 3 Department of Family Medicine, Medical University of Bialystok, ul Mieszka I 4B, 15-054 Bialystok, Poland; 4 Department of Foreign Languages, Medical University of Bialystok, ul. Mickiewicza 2C, 15-222 Bialystok, Poland; 5 Faculty of Engineering Management, Bialystok University of Technology, ul. Ojca Tarasiuka 2, 16-001 Kleosin, Poland; 6 School of Human Sciences, University of Economics and Human Sciences in Warsaw, ul. Okopowa 59, 01-043 Warsaw, Poland; alexandramacdonald02@gmail.com; 7 Non-Public Specialist Medical Practice Adrian Wojciech Przystupa, ul. Aleja Józefa Piłsudskiego 31, 17-100 Bielsk Podlaski, Poland

**Keywords:** health-related quality of life, KIDSCREEN-27, high myopia, adolescents

## Abstract

**Background/Objectives:** The aim of the study was to explore Health-Related Quality of Life (HRQoL) using the KIDSCREEN-27 questionnaire among adolescents with high myopia (HM). **Methods**: Sixty-nine adolescents with HM and 71 healthy participants aged 12–17 years and their parents or legal guardians were enrolled in the study. **Results:** Adolescents with HM showed significantly lower scores on the Physical Well-Being dimension in comparison with controls (*p* = 0.003), particularly girls with HM in comparison with girls from the control group (*p* = 0.008), and 15–17-year-old adolescents in comparison with same-aged controls (*p* = 0.020). Girls with HM were characterised by significantly worse scores on the Psychological Well-Being dimension compared with boys with HM (*p* < 0.042). Sociodemographic factors and refractive error, its duration, and acceptance of disease had no impact on HRQoL. **Conclusions:** HM may have a negative impact on the HRQoL of children, affecting particularly the physical and psychological well-being of girls. It is important that a holistic approach to the treatment of HM in adolescents is taken by measuring their HRQoL as part of the routine diagnostic process. Use of the KIDSCREEN-27 questionnaire seems justified as it allows for determination of the type of intervention required to improve the HRQoL of individuals affected by the disease.

## 1. Introduction

Myopia, particularly high myopia (HM) defined as a spherical equivalent of ≤−6.00 Dioptres (D), has come into focus as a worldwide public health concern because of its growing incidence, onset at a younger age and more rapid progression. Its prevalence is higher in Asia compared with Europe. Myopia is commonly detected before the age of 10, but onset can vary from 3 to 4 years of age into late adolescence or early adulthood, depending on ethnic, familial, environmental and geographic factors [1].

In recent years, Health-Related Quality of Life (HRQoL) has attracted much research attention. HRQoL is a multidimensional construct that includes physical, emotional, mental, social and behavioural components of well-being and function as perceived by patients and/or other individuals [2]. The WHO Quality of Life Group expands this definition and incorporates a cultural perspective: quality of life is defined as an individual’s perception of their position in life in the context of the culture and value systems in which they live, and in relation to their goals, expectations, standards and concerns [3]. HRQoL combines the person’s past experience with their current situation and hopes for the future. It is a measure of the discrepancy between the individual’s aspirations (what they desire and want to achieve) and their current situation: the more compatible they are, the higher the person’s quality of life (QoL) [4]. It should be emphasised that HRQoL is a subjective assessment and expression of well-being, and therefore differs from person to person as the criteria applied in the evaluation are not objective. The individual’s perception of their QoL is affected by a system of goals, values and beliefs developed during the process of identity formation. Adolescence is a crucial period in this process—a young person strives to establish who they are and who they want to be in the future, their role in society and their relationships with others. According to Erikson’s psychosocial theory of development [5,6], identity provides a frame of reference for interpreting one’s own personality. Angus Campbell was a pioneer and author of extensive psychological research into QoL, which he defined as the level of satisfaction with individual areas of life expressed in reflective assessments of these areas [7].

Ophthalmologists and other healthcare professionals are aware of the importance of patient-centred care—a holistic model of care that respects the patient’s experiences, values, beliefs and preferences in the planning, management and delivery of care. Over the past decades, a paradigm shift has also occurred in evaluating healthcare outcomes. The focus has moved from clinical indicators of disease activity to the patient’s perception of their health condition and relevant treatment. The concept of HRQoL has been introduced and, along with morbidity and mortality, is recognised as one of the basic measures of evaluating the health of the population.

Assessment of QoL in children and adolescents depends on the availability of appropriate research instruments that take into consideration the child’s age, maturity and their cognitive abilities. Such instruments can help detect deterioration in QoL early and allow for prompt intervention. In recent years, several instruments for children with visual impairment (VI) have been developed [8,9,10]. In our investigation, we used the Polish version of the KIDSCREEN-27 questionnaire [11] to assess HRQoL in adolescents. Our previous study found that myopia may have an impact on trait anxiety among 13–14-year-olds [12]. Therefore, we hypothesised that the HRQoL reported by adolescents with HM would be different from the HRQoL reported by healthy adolescents. It should be emphasised, however, that the objectively measured health condition is not in itself a determinant of QoL since objective factors, e.g., myopia, are interpreted subjectively.

The aim of the study was to measure HRQoL among adolescents with HM using the validated KIDSCREEN-27 questionnaire and to examine factors determining the scores on individual HRQoL dimensions. The following determinants were explored in the study: sociodemographic factors (gender and age, parents’ marital status and education level) and refractive error, its duration, and acceptance of disease among adolescents with HM.

## 2. Materials and Methods

A cross-sectional study was conducted between February and December 2022 among adolescents with HM who were recruited during a routine check-up at the Department of Paediatric Ophthalmology and Strabismus at the University Children’s Hospital in Bialystok. The control group was selected from the electronic database and medical records of a General Practitioner’s (GP) surgery in Bialystok. Age- and gender-matched healthy adolescents were recruited using a random number table. Written informed consent for study participation was obtained from the participants or legal guardians of subjects under 16 years of age. The study was conducted in accordance with the Declaration of Helsinki Guidelines for Biomedical Research Involving Human Subjects. The study was approved by the local Ethics Committee of the Medical University of Bialystok, Poland (No. APK.002.77.2020).

### 2.1. Study Population

The study included 69 HM adolescents aged 12–17 years and their parents or legal guardians aged 30–61 years. The control group consisted of 71 healthy adolescents without myopia, with hyperopia from +0.25 D to a maximum of +1.25 D, aged 12–17 years, and their parents aged 32–59 years.

Adolescents with comorbid eye conditions, including syndromic myopia (e.g., retinal dystrophies, Stickler syndrome), a history of treatment for retinopathy of prematurity, or additional disabilities such as hearing, cognitive, mental or behavioural disorders, were excluded from study participation.

The inclusion criterion for children with HM was the myopic spherical equivalent ≤ −6.0 D and normal intraocular pressure in the range of 14–18 mmHg. Only children with a best-corrected visual acuity (BCVA) of 0.8 (20/25) or better in each eye were included.

Children completed HRQoL questionnaires (KIDSCREEN-27) in face-to-face interviews. Parents also completed questions regarding sociodemographic and medical characteristics of their child.

### 2.2. Instruments

#### 2.2.1. Health-Related Quality of Life Questionnaire (KIDSCREEN-27)

We used the Polish version of the KIDSCREEN-27 questionnaire to assess HRQoL because of its excellent psychometric properties. It is a shorter version of the KIDSCREEN-52 questionnaire—its completion takes approximately 10 to 15 min, thus reducing the time burden on the participants. The tool has been found to have excellent cross-cultural comparative validity [11,13,14]. The principle adopted in the questionnaire is that adolescents are asked questions about their feelings over the last week. The KIDSCREEN-27 questionnaire consists of 27 items and measures the following five dimensions:

- Physical Well-Being (5 items)—level of physical activity, energy and fitness of the adolescent, health and complaints.
- Psychological Well-Being (7 items)—positive and negative emotions, self-perception, self-esteem, worries and stress, life satisfaction and optimism.
- Autonomy and Parent Relation (7 items)—autonomy, home life and parents, and quality of financial resources perceived by the adolescent.
- Peers and Social Support (4 items)—quality of the adolescent’s social relations and interactions with friends and peers, and the degree of their perceived social support.
- School Environment (4 items)—the adolescent’s perception of their cognitive capacity, concentration and learning in the school environment. The adolescent’s view of their relationship with teachers.

Answers to the KIDSCREEN-27 questionnaire were provided using standardised 5-level categories, with ratings ranging from 0 to 4, indicating either frequency (never/seldom/quite often/very often/always) or intensity (not at all/slightly/moderately/very/extremely). The first question, concerning general health, utilised a different scale, defining health as excellent/very good/good/fair/poor. Certain items (1, 9, 10 and 11) were reversed during scoring as per standard performance procedures, as higher numbers originally indicated a worse score. Responses to these items were reversed to maintain consistency across the questionnaire. A maximum of 108 points could be obtained for the questionnaire (a maximum of 20 points for the first dimension, a maximum of 28 points for the second and third dimensions, and a maximum of 16 points for the fourth and fifth dimensions). In order to standardise the results, self-reported scores in individual dimensions were converted using a 0–100 point scale (questions scored from 0 to 4, sum of points divided by the maximum for a given dimension and multiplied by 100). We decided to present the data as S-scores because the natural distinctions between the results obtained for individual partial dimensions of QoL are preserved. Higher scores indicate better HRQoL.

#### 2.2.2. Sociodemographic Factors and Factors Related to HM

An original questionnaire was used to collect information regarding the sociodemographic characteristics of study participants, including the adolescent’s gender and age, parents’ marital status and parental educational attainment.

A 5-point Likert scale ranging from ‘1’ to ‘5’ (strongly disagree/disagree/neither agree not disagree/agree/strongly agree) was used to assess acceptance of disease by the adolescents [15].

The parents of the adolescents with HM were also asked about their children’s refractive error and disease duration.

#### 2.2.3. Ophthalmoscopic Examination

Visual acuity and intraocular pressure were measured in all adolescents (n = 140). In the adolescents with HM, refractive error was examined after paralysis of accommodation with 1% Tropicamide, instilled three times at 15-min intervals using TONOREF™ Nidek’s Auto Ref/Kerato/Tono/Pachymeter. Children underwent a slit-lamp examination in order to exclude conditions other than high myopia, including syndromic myopia (e.g., retinal dystrophies, Stickler syndrome), and those with a history of treatment for retinopathy of prematurity.

#### 2.2.4. Statistical Methods

Student’s *t*-test was used to compare the scores on five KIDSCREEN dimensions (expressed as S-scores) between the groups of adolescents with HM and healthy controls and between the subgroups depending on gender and age. The strength of interdependence between them was assessed using Pearson’s correlation coefficient. In the group of children with HM, refractive error was determined in the right and left eye. Differences between the right and left eye were insignificant, and therefore we decided to use a variable defined as a refractive error (the lowest selected from both eyes). The Mann–Whitney U test was used to assess differences in refractive error in adolescents with HM. Spearman’s correlation coefficient was used to assess correlations between refractive error and disease duration and between refractive error and other parameters. Correlations and differences were considered statistically significant at *p* < 0.05.

Two clusters of adolescents with HM were generated based on the scores on five dimensions: Physical Well-Being, Psychological Well-Being, Autonomy and Parent Relation, Peers and Social Support, and School Environment. The first cluster included adolescents who reported higher S-scores on these dimensions, and the second included adolescents who reported lower scores on these dimensions.

## 3. Results

In the group of teenagers with HM, the median of refractive error was −6.75 D, with quartiles Q1 = −6.00 D and Q2 = −8.75 D, and the median of disease duration was 9 years and ranged from 2 to 17 years. A significant negative correlation was found between disease duration and refractive error (r = −0.4159, *p* < 0.0004). Basic characteristics of both groups (HM and controls) and their parents are presented in Table 1.

There were no significant differences in the girls/boys ratio between the two groups (adolescents with HM and controls) (*p* < 0.601) or between the subgroups of parents (*p* < 0.484). There were also no significant age differences between adolescents with HM and controls (*p* < 0.858) or between the two groups of parents (*p* < 0.487). In the control parent group, parental marital status was reported as ‘other’ more frequently (*p* < 0.032), and a significantly larger number of parents had higher education (*p* < 0.042) in comparison with the group of parents of HM children.

### 3.1. Comparative Analysis of Child-Reported KIDSCREEN-27 Dimension Scores

Adolescents with HM exhibited significantly lower scores on the Physical Well-Being dimension in comparison with the control group (*p* = 0.003) (Table 2). There were no significant differences between the scores on the remaining four dimensions when comparing the two groups.

Analysis of gender subgroup scores showed that girls with HM reported significantly lower scores on the Psychological Well-Being dimension in comparison with boys with HM (*p* < 0.042). There were no significant differences between the scores on the remaining four dimensions when comparing the two groups. In the control group, no significant gender differences were observed in the scores across the dimensions (Table 3).

Analysis revealed that girls with HM reported lower scores on the Physical Well-Being dimension compared with girls from the control group (** *p* = 0.008) (this *p* is not shown in Table 3). When the subgroups of boys with HM and boys from the control group were compared, it was found that there were no differences in the scores across the dimensions.

Analysis of age subgroup scores (12–14-year-olds versus 15–17-year-olds) of adolescents with HM and the controls (Table 4) showed that there were no significant differences between the subgroups across the dimensions.

Comparisons were also made between the subgroups of 12–14-year-old children with HM and 12–14-year-old controls. It was found that 12–14-year-olds with HM reported lower scores on the Physical Well-Being dimension (at the border of statistical significance) compared with same-aged controls (*** *p* < 0.061) (this *p* is not shown in Table 4). There were no significant differences between the scores on the remaining four dimensions when comparing the two groups.

The same comparative analysis was performed between the subgroups of 15–17-year-old adolescents with HM and same-aged controls. It was established that 15–17-year-olds with HM reported lower scores on the Physical Well-Being dimension in comparison with same-aged controls (*** *p* = 0.020) (this *p* is not shown in Table 4). There were no significant differences between the scores on the remaining four dimensions when comparing the two groups.

### 3.2. Analysis of KIDSCREEN-27 Dimension Scores Reported by Adolescents with HM

Correlation coefficients between the KIDSCREEN-27 dimension scores reported by adolescents with HM are positive and statistically significant (Table 5).

**Figure 1:**
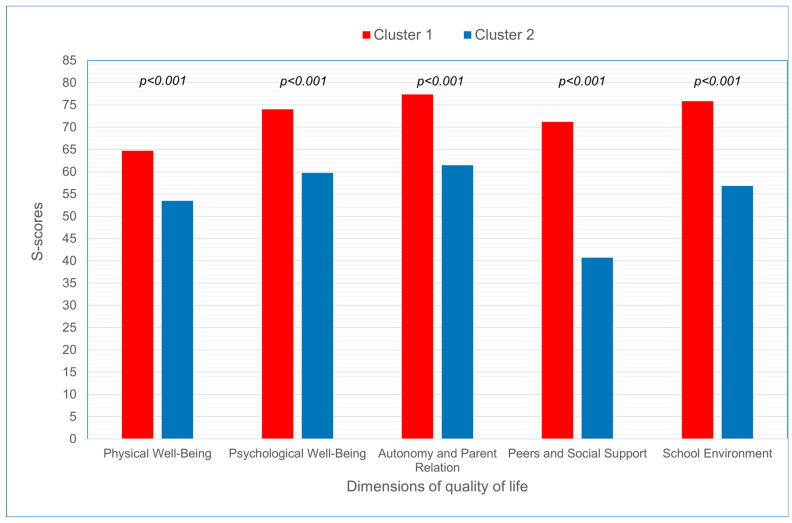
S-scores for KIDSCREEN-27 dimensions reported by adolescents with HM in cluster 1 and in cluster 2.

In order to examine the effect of sociodemographic factors (gender, age, parents’ marital status and parental educational attainment) and refractive error, its duration, and acceptance of disease on KIDSCREEN-27 dimension scores reported by children with HM, cluster analysis was performed. Two clusters, as distant from each other as possible and internally coherent, were generated. Significantly higher scores were observed across all KIDSCREEN-27 dimensions in the first cluster in comparison with the second cluster (*p* < 0.000) (Figure 1, Table 6).

It was revealed that sociodemographic factors and refractive error, its duration, and acceptance of disease had no impact on any of the KIDSCREEN-27 dimension scores reported by children with HM (Table 7).

## 4. Discussion

The study examined the HRQoL of 12–17-year-old adolescents with HM measured using the standardised KIDSCREEN-27 questionnaire. We found mixed results for two KIDSCREEN-27 dimensions. It was revealed that adolescents with HM scored significantly worse on the Physical Well-Being dimension in comparison with the control group. When looking at gender and age subgroup scores, girls and 15–17-year-old adolescents with HM reported significantly lower scores on the Physical Well-Being dimension in comparison with control subgroups. Differences were also observed in the group of 12– 14-year-old children with HM in comparison with same-aged controls, but they were at the border of statistical significance. Furthermore, girls with HM reported significantly lower scores on Psychological Well-Being compared with boys with HM. No significant differences in the scores on the remaining three dimensions (Autonomy and Parent Relation, Peers and Social Support and School Environment) were found between the subgroups of children.

Since our investigation is the first one to evaluate HRQoL in a homogeneous group of adolescents with HM using the KIDSCREEN-27 questionnaire, it is difficult to compare our results with the findings of other authors. Nonetheless, our findings are inconsistent with the results of the only study conducted using the KIDSCREEN-27 questionnaire among children and adolescents with visual impairment (VI) [16]. In the cited study, boys and girls aged 12–17 years with VI reported similar scores on the Physical Well-Being dimension in comparison with boys and girls from the control group. It must be noted that the study included children and adolescents with VI caused by various eye diseases, whose common feature was VI, understood as having best-corrected visual acuity < 0.3, visual field < 30 degrees, in the case of disorders in lower or higher visual functions (e.g., respectively, night blindness/photophobia or cerebral VI), or in the case of a progressive disorder, or in the case of rehabilitation need for which no opportunities in regular ophthalmological care exist. It must be emphasised that in our investigation HM was a criterion for inclusion in the study group, and, despite the degenerative nature of the disease, individuals may have full visual acuity, with no visual field limitations or other visual impairments if correction is properly selected.

Our results are consistent with the results of other studies in which children and young adults with VI often scored significantly worse than peers with normal vision on the subscales of QoL [17,18,19,20]. Some investigations have confirmed that children with VI are less physically active, have poorer physical fitness and lead more sedentary lifestyles compared with children with no reported VI [16,21].

Our study revealed significant differences in the scores on the Psychological Well-Being dimension between girls with HM and boys with HM. In contrast, Elsman et al. observed a considerably higher level of psychological well-being in girls with VI (aged 12–17 years) compared with same-aged female controls. Boys with VI (aged 12–17) reported a comparable level of psychological well-being with boys from the control group [16].

Our findings align with a common trend that men tend to view themselves more positively than women. Men often exhibit greater contentment with their physical attractiveness and are less preoccupied with perceived attractiveness compared with women [22]. Furthermore, research by Buss showed that in 34 out of 37 samples of cross-cultural data males valued physical attractiveness in potential mates more than females did [23].

Dias et al. demonstrated that contact lens wearers, compared with eyeglass wearers, were more likely to be females and had higher self-esteem [24]. Wearing contact lenses instead of’ ‘unsightly’ glasses may increase girls’ self-esteem, and thus psychological well-being, as they are more preoccupied with physical attractiveness than boys. It should be emphasised that adolescence is a critical period not only in the physical but also mental development of an individual. The perception of spectacle wearers as ‘weaker’ in some cultures may lead to higher levels of anxiety among adolescents with reduced visual acuity. Young people with myopia are more likely to suffer from anxiety or depression in comparison with their peers without myopia [12]. Patients with chronic diseases commonly report feelings of worry, sadness and apprehension, which may lead to the development of anxiety or depressive disorders [25], as confirmed by studies among individuals with VI in comparison with those without VI [26,27].

In the case of children and adolescents, an important process is the formation of the Self. Individuals tend to choose experiences and situations that align with their personal beliefs about themselves. Children and adolescents who perceive themselves to be visually impaired behave differently from those who consider themselves healthy. Many of these perceptions and beliefs arise relatively early and may act as self-fulfilling prophecies, and thus shape, as Bee notes, the trajectory of a young person’s life [28]. Jastrzębski and Pasiak have demonstrated a positive relationship between self-esteem and QoL [29]. People with low self-esteem often experience negative emotions, rarely face challenges, are unable to overcome difficulties, lack perseverance to accomplish tasks they have undertaken and downplay their achievements. People with high self-esteem experience positive emotions, are energetic and dynamic, rise to challenges and are more likely to overcome difficulties [30].

It is worth noting that HRQoL is affected by several other factors, e.g., personality [31]. A strong positive relationship has been found between HRQoL and extraversion (a strong need for stimulation) and conscientiousness (the quality of working hard, and being careful and precise), whereas a negative relationship has been established between HRQoL and neuroticism (heightened sensitivity to difficulties). Factors affecting QoL also include a sense of control over one’s own development (the life-span theory of control, Heckhausen and Schulz), action orientation (Kuhl), interpersonal competence (Plopa) and the level of self-awareness (Wicklund) [32].

In our study, we found no differences in the scores on the Autonomy and Parent Relation, Peers and Social Support, and School Environment dimensions between the group of adolescents with HM and the controls. We also did not reveal any differences in the scores on the three dimensions within the group with HM or within the control group when the criteria of gender and age were applied. Contradictory results were obtained by Elsman et al. who demonstrated that adolescents with VI reported better scores on the Autonomy and Parent Relation (both boys and girls aged 12–17 years) and School Environment (only boys aged 12–17 years) dimensions in comparison with same-aged healthy controls. Self-reported scores on the Peers and Social Support dimension were comparable in boys and girls with VI and controls [16]. Empirical research indicates that children with VI have fewer friends, perceive the quality of their friendships as lower, are part of smaller social networks and report a sense of loneliness more frequently than their sighted peers [33,34,35,36]. Similarly to our study, Banaszkiewicz and Żurek demonstrated that boys reported significantly better levels of mental well-being, had a more positive self-image and were happier with their friendships in comparison with girls. Social aspects of HRQoL were assessed similarly by both genders [37].

Our study revealed that worse scores on the Physical Well-Being dimension were associated with worse scores on other dimensions (positive correlation coefficients). Studies by other authors have confirmed the relationship between physical and psychological well-being, and have suggested that participation in sport and physical activity and reducing sedentary behaviours might protect children’s mental health [38]. Intervention of physical activity alone is associated with increased self-concept and self-worth in children and adolescents [39]. On the other hand, there is some evidence that physical activity might be protective against myopia development, and may help to stabilise visual acuity without correction [40,41]. From a theoretical standpoint, it seems reasonable to formulate a hypothesis that increased choroidal blood perfusion may inhibit the progression of myopia. Research on animals indicates that increased choroidal blood perfusion attenuates scleral hypoxia and thereby inhibits the development of myopia in guinea pigs [42].

Cluster analysis of the adolescents with HM performed in our study revealed two clusters—one with better and one with worse scores on the KIDSCREEN-27 dimensions. It was demonstrated that sociodemographic factors (age and gender, parents’ marital status and their educational level) and refractive error, its duration, and acceptance of disease by the adolescents were not factors differentiating self-reports on the five KIDSCREEN-27 dimensions. Similar results were obtained by Elsman et al. who reported no significant trends towards worse HRQoL in young adults with more severe VI [16,17]. Furthermore, a study by Wong HB et al. showed that refractive errors did not appear to have an impact on the QoL of adolescents [18]. By contrast, other authors have demonstrated that disease duration and the extent of vision loss, rather than socioeconomic factors and comorbidities, affect vision-related QoL [43].

The study has several limitations, including a small sample size. Our aim was to conduct a study in a homogeneous group—among children with HM. Therefore, we had to conduct multi-stage diagnostic tests in a hospital setting which were time-consuming and costly, but allowed for a precise selection of study participants. A potential limitation of this study is the risk of selection bias, as participants were recruited from patients attending ophthalmology clinics. This might have led to a higher participation rate among individuals more concerned with their eye health. Additionally, the duration of high myopia was reported by parents, which might not always align with clinical records. Future research should aim to include a more representative sample and incorporate verified clinical data to enhance the robustness of the findings. The KIDSCREEN-27 questionnaire was used to assess QoL, which is not a standard tool for diagnosing visual disorders, but it is a modern, validated instrument assessing aspects of the physical, mental and social health of teenagers.

## 5. Conclusions

The results of the study indicate that HM may have a negative impact on the HRQoL of adolescents, affecting particularly the physical and psychological well-being of girls. Therefore, it is important that a holistic approach to the treatment of HM in young people is taken by measuring their HRQoL as part of the routine diagnostic process of the disease.

It is assumed that the individual life course is determined by the person’s biological and psychological characteristics, and the properties of a widely understood environment. As demonstrated by Steuden and Oleś, loss of visual function is not only a medical problem but also a psychological one [44]. Use of the KIDSCREEN-27 questionnaire to measure HRQoL among adolescents with HM seems justified as it allows for determination of the type of intervention required to improve the quality of life of individuals affected by the disease.

## Figures and Tables

**Table 1:** Basic characteristics of adolescents with high myopia (HM), controls, and their parents.

Sociodemographic Factors		High Myopia		Control Group	
		Adolescents, n (%)	Parents, n (%)	Adolescents, n (%)	Parents, n (%)
Number		69 (100)	69 (100)	71 (100)	71 (100)
Gender	Female	39 (56.5)	52 (75.4)	37 (52.1)	57 (80.3)
	Male	30 (43.5)	17 (24.6)	34 (47.9)	14 (19.7)
Age subgroup	Adolescents 12–14 years	32 (46.4)		34 (48.0)	
	Adolescents 15–17 years	37 (53.6)		37 (52.0)	
	Parents < 42 years		29 (42.0)		34 (48.0)
	Parents ≥ 42		40 (58.0)		37 (52.0)
Parents’ marital status	Married		64 (92.7)		57 (80.3)
	Other		5 (7.3)		14 (19.7)
Education	Primary	43 (62.3)	17 (24.6)	57 (80.3)	13 (18.3)
	Secondary	26 (37.7)	25 (36.2)	14 (19.7)	18 (25.4)
	Higher		27 (39.2)		40 (56.3)

**Table 2:** Child-reported KIDSCREEN-27 dimension scores.

Dimension	Control Group, n = 71	High Myopia, n = 69	*p* Values <
Physical Well-Being	66.90 ± 15.77	59.35 ± 13.23	0.003 *
Psychological Well-Being	68.66 ± 13.17	67.18 ± 13.55	0.514
Autonomy and Parent Relation	69.01 ± 15.54	69.77 ± 14.16	0.764
Peers and Social Support	54.49 ± 21.16	56.61 ± 20.45	0.547
School Environment	64.88 ± 15.03	66.76 ± 15.95	0.474

Mean ± standard deviation (S-score). * Statistically significant with a *p*-value of <0.05.

**Table 3:** Child-reported KIDSCREEN-27 dimension scores by gender.

Dimension	High Myopia		*p* Values <
	Girls, n = 39	Boys, n = 30	
Physical Well-Being	59.10 ± 12.77 **	59.67 ± 14.02	0.863
Psychological Well-Being	64.29 ± 14.03	70.95 ± 12.11	0.042 *
Autonomy and Parent Relation	68.59 ± 13.59	71.31 ± 14.96	0.434
Peers and Social Support	57.21 ± 19.53	55.83 ± 21.89	0.784
School Environment	67.47 ± 16.49	65.83 ± 15.46	0.677
	Control group		
	Girls, n = 37	Boys, n = 34	
Physical Well-Being	67.70 ± 14.51	66.03 ± 17.22	0.659
Psychological Well-Being	67.95 ± 13.05	69.44 ± 13.46	0.640
Autonomy and Parent Relation	67.66 ±15.08	70.48 ± 16.12	0.449
Peers and Social Support	54.56 ± 19.19	54.41 ± 23.41	0.977
School Environment	63.18 ± 15.99	66.73 ± 13.91	0.323

Mean ± standard deviation (S-score). *, ** Statistically significant with a *p*-value of < 0.05.

**Table 4:** Child-reported KIDSCREEN-27 dimension scores by age.

Dimension	High Myopia		*p* Values <
	12–14 years, n = 32	15–17 years, n = 37	
Physical Well-Being	60.00 ± 12.38	58.78 ± 14.06 ***	0.707
Psychological Well-Being	68.30 ± 13.75	66.22 ± 13.48	0.528 *
Autonomy and Parent Relation	69.31 ± 15.42	70.17 ± 13.18	0.803
Peers and Social Support	52.54 ± 19.37	60.14 ± 20.96	0.125
School Environment	64.45 ± 18.81	68.75 ± 12.93	0.268
	Control group		
	12–14 years, n = 34	15–17 years, n = 37	
Physical Well-Being	67.06 ± 17.15	66.76 ± 14.64	0.937
Psychological Well-Being	67.75 ± 14.59	69.50 ± 11.86	0.581
Autonomy and Parent Relation	67.44 ± 16.09	70.46 ± 15.10	0.416
Peers and Social Support	52.76 ± 18.35	56.08 ± 23.59	0.512
School Environment	62.32 ± 14.56	67.23 ± 15.27	0.170

Mean ± standard deviation (S-score). *, *** Statistically significant with a *p*-value of < 0.05.

**Table 5:** Correlation coefficients between the KIDSCREEN-27 dimension scores reported by adolescents with HM (n = 69).

Dimension	Dimension	r	*p* Values <
Physical Well-Being	Psychological Well-Being	0.4548	0.0001
	Autonomy and Parent Relation	0.3587	0.0025
	Peers and Social Support	0.3391	0.0044
	School Environment	0.4599	0.0001
Psychological Well-Being	Autonomy and Parent Relation	0.4663	0.0001
	Peers and Social Support	0.4571	0.0001
	School Environment	0.5800	0.0000
Autonomy and Parent Relation	Peers and Social Support	0.3569	0.0026
	School Environment	0.4632	0.0001

**Table 6:** S-scores for KIDSCREEN-27 dimensions reported by adolescents with HM in cluster 1 and in cluster 2.

Dimension	Cluster 1	Cluster 2
	n = 36	n = 33
Physical Well-Being	64.72 ± 12.07	53.49 ± 12.02
Psychological Well-Being	74.01 ± 11.63	59.74 ± 11.51
Autonomy and Parent Relation	77.38 ± 10.97	61.47 ± 12.59
Peers and Social Support	71.18 ± 14.27	40.72 ± 12.90
School Environment	75.87 ± 12.24	56.82 ± 13.47

Mean ± standard deviation (S-score).

**Table 7:** Differences in sociodemographic factors (adolescents and their parents) and refractive error, its duration, and acceptance of disease between adolescents with HM in cluster 1 and in cluster 2.

Variables, n (%)	Cluster 1	Cluster 2	*p* Values <
Gender (boys/girls)	16 (44.4)/20 (55.6)	14(42.4)/19 (57.9)	0.866
Age subgroup (12–14/15–17 years)	14 (38.9)/22 (61.1)	18 (54.6)/15 (45.4)	0.193
Parents’ education (higher/other)	14 (38.9)/22 (61.1)	13 (39.4)/20 (60.6)	0.966
Parents’ marital status (married/other)	33 (91.7)/3 (8.3)	31 (93.9)/2 (6.1)	0.717
Refractive error (≤−7/>−7 Dioptre)	18 (50.0)/18 (50.0)	16 (48.5)/17 (51.5)	0.900
Duration of disease <10/≥10 years	18 (50.0)/18 (50.0)	17 (51.5)/16 (48.5)	0.900
Acceptance of disease 1–3/4–5 points	17 (47.2)/19 (54.3)	17 (51.5)/16 (48.5)	0.722

## Data Availability

The data are available on request from the authors.
